# Supplementary material for: Locomotion in virtual environments predicts cardiovascular responsiveness to subsequent stressful challenges
Source: Nat Commun. 2020 Nov 19;11:5904. doi: 10.1038/s41467-020-19736-3 (PMC7677550; doi:10.1038/s41467-020-19736-3)
Supplement: Supplementary file 1 — Supplementary Information [file 41467_2020_19736_MOESM1_ESM.pdf]

## Supplementary Information

# Locomotion in virtual environments predicts cardiovascular responsiveness to subsequent stressful challenges

João Rodrigues[a]*, Erik Studer[a], Stephan Streuber[a], Nathalie Meyer[a] and Carmen Sandi[a]*

(a) Laboratory of Behavioral Genetics, Brain Mind Institute, School of Life Sciences, École Polytechnique Fédérale de Lausanne, EPFL, Lausanne 1015, Switzerland

* Correspondence: carmen.sandi@epfl.ch; joao.rodrigues@epfl.ch

## Contents

**Supplementary Notes** .......... 2

**Supplementary Methods**

- Participants .......... 3
- Cardiorespiratory variables .......... 3
- Behavioral feature description .......... 4

**Supplementary Results** .......... 7

- Statistics for physiology in exploration scenarios: Dark maze .......... 7
- Statistics for physiology in exploration scenarios: Empty room .......... 8
- Statistics for physiology in exploration scenarios: Elevated alley .......... 8
- PCA compression for cardiorespiratory variables .......... 9
- Model validation on the training dataset .......... 10
- Selected features .......... 11
- XGBoost algorithm .......... 11
- Statistics for physiology in Fig. 4b .......... 15
- Physiology analysis .......... 16
- Stress test .......... 17
- Sustained anticipatory anxiety paradigm: Fear conditioning test .......... 17

**Supplementary References** .......... 20

# Supplementary Notes

The heart is innervated by preganglionic sympathetic and parasympathetic neurons respectively via the stellate ganglia and the vagus nerve [1–4]. Despite this dual innervation, it is the parasympathetic nervous system that dominates this organ's activity by exerting a continuous inhibitory control over an intrinsically higher resting heart rate (HR) [5,6]. This vagal influence is characterized by fast responses (reductions in HR peaking after 0.5 s and returning to baseline after 1 s) which are able to produce beat-to-beat changes in the HR; something that the slower sympathetic influence (increases in HR peaking after 4 s and returning to baseline after 20 s) cannot replicate [7–9].

These fast beat-to-beat changes and the slower sympathetic influences in HR are what the heart rate variability (HRV), with its multiple formulations, attempts to capture: a low HRV is a consequence of more constant inter beat intervals (IBIs) while a high HRV results of more variable intervals between heartbeats. Several formulas have been used to compute HRV, ranging from metrics more favorable to long-term recordings (ex: the approximate entropy of all IBIs during 24 h) to metrics more adequate for shorter recording. These can be either sensitive to overall HR variability like the standard deviation, or more appropriate to capture the beat-to-beat parasympathetic effects, like the root mean square of successive differences (RMSSD). In addition, power spectral analysis of IBI time series has frequently been used to quantify HRV with high frequency (HF-HRV) (0.15–0.40 Hz) and low frequency (LF-HRV) (0.01–0.15 Hz) reflecting cardiac vagal tone and a mixture of vagal and sympathetic influences, respectively. The latter is better represented by the electrodermal activity (EDA) which is considered a purely sympathetic metric [10].

Higher HRV has been associated with a general adaptive responsiveness to changes in not only the internal environment (e.g., protecting from high trait anxiety effects in attention to threat [11], state anxiety in healthy individuals [12] and worry [13]) but also in the external environment (initiate and maintain positive social relations [14], sensitivity to others emotional states [15]).

Lower HRV has been associated with a decreased ability to respond flexibly [e.g., compromised ability to recognize safety in patients with anxiety disorders [16]]. Low resting HRV has been proposed as a marker for disease [17] and has been associated with increased risk of all-cause mortality [18,19]. On the other hand, having high resting HRV has been associated with higher emotional well-being [20,21], with lower levels of worry and rumination [13,22], lower anxiety [23], and better regulated emotional responding [24].

HRV extracted from pulse oximeters [pulse rate variability (PRV)] is rapidly being introduced as a feature in commercial products like smart watches and wristbands as an index of stress exposure and energy [25]. However, PRV and HRV are not always correlated, especially during mental stress or physical activity [25] but have been found to be highly correlated in healthy individuals during rest [26].

However, an increasing number of studies has been suggesting HRV to be more than just an index of healthy heart function, by indexing how a network of brain regions responsible for adaptive regulation provide adaptive control over the periphery and allowing the organism to effectively function in a complex environment. Low resting HF-HRV and large reductions in this metric to assorted challenges are associated with symptoms of both internalizing and externalizing psychopathology [21,27,28], and with a wide range of psychopathological syndromes, including anxiety [29,30], phobias [31], callousness [32], conduct disorder [33], depression [34–36], trait hostility [37], psychopathy [38], among others. Furthermore, HRV reactivity has been suggested as a reliable index of the ability to

self-regulate and react to stress in the environment [19,39] and HRV reactivity to social stressors may be particularly important in predicting depression risk during adolescence [40].

The use of computer simulated immersive virtual environments (IVEs) in research has been increasing in popularity over the last decade not only due to its increased availability but most importantly due to the initial pioneering experiments that shed the necessary light on its potential applications. IVEs place the user in an interactive virtual reality (VR) that can be programmed to deliver standardized and controlled manipulations making them an attractive technique in several life-science fields. As an emotive medium, IVEs have been effectively used to influence self-reported emotions like anxiety and relaxation [41] or elicit self-reported anger and physiologic arousal [42]. There is also considerable evidence that IVEs can be used to create stress induction tasks that effectively elicit psychophysiological stress responses in both clinical and non-clinical samples. For instance, it has been shown that modifying the controllability of the vestibular system in a dynamic IVE increased cortisol secretion compared to a static IVE [43], or that owning a VR body in an uncomfortable position also leads to increasing markers of stress [44]. Psychosocial stress related protocols have also been used in VR showing that anticipating public speaking in an IVE increased subjective anxiety and physiological responses in HR, EDA and startle reactivity [45]. One important aspect revealed by aforementioned IVE studies is that these environments can also be used to separate individuals with different psychophysiological phenotypes using less conspicuous cues.

# Supplementary Methods

## Participants

135 participants were admitted to the study. Criteria for inclusion in the study included being healthy, male, 18 – 38-year-old, right-handed, French-speaking, non-smoking, no history of neurological, psychological or cardiac disease and no corrected vision. Exclusion criteria consisted of self-reported psychiatric, neurological and medical conditions. After receiving a complete description of the study, each participant gave informed consent to participate. Participants were given a financial compensation of 20 CHF per hour. Experimental sessions were scheduled between 1 pm and 7 pm. The study was approved by the Swiss Cantonal Commission for Ethics of Human Research in the Canton of Vaud (CER-VD).

## Cardiorespiratory variables

HRV is known to be dominated by parasympathetic (vagal) influence, which affects heart rate with short latencies around 0.5 s. In turn, sympathetic influence has a slower influence, peaking approximately within 4 s and returning to baseline after 20 s.

HR was computed from the electrocardiogram (ECG) time-series (recorded wirelessly with a Biopac BioNomadix® unit), which was sampled at 1kHz and decimated to 500 Hz. These conditions allow the identification of the QRS complex with high precision, which requires sampling rates no lower than 250 Hz [46,47]. We used a Pan Tompkins algorithm [48] to detect the QRS complexes. After visual inspection for potential ECG artifacts or ectopic beats, the identified R peaks were used, and the R to R intervals were taken to create an IBI time-series. This was then processed for outliers with a standard deviation filter of 2.5. Detected outliers were removed and the neighboring R to R intervals used for linear interpolation.

HRV measures the temporal changes in HR beat after beat and has been formulated in several different ways, from time- and frequency-domain methods as well as geometric and non-linear methods [47]. Here, we chose to use formulas that can be applied in short time intervals, ultra-short HRV [49–51], but avoided using spectral HRV measurements due to their sensitivity to the spectral computation method and choice of time window [52]. Since the high-frequency HRV (HF-HRV) component is known to represent parasympathetic activity with good specificity [53], and the root mean square of successive differences (RMSSD) is highly correlated with it [19] and less affected by respiration than HF-HRV [54,55], we chose the latter time-domain formula for our analyses. Additionally, we used two other time-domain formulas that have been used with short length ECG data [51]: standard deviation of the R to R intervals (SDNN) and the triangular index (HRVTi), the latter highly investitive to artifacts [56].

HRV can be affected by breathing patterns [56] and in this experiment we did not ask participants to perform controlled breathing. Although the RMSSD formula is less affected by respiration than other indices of HRV [54,55], we recorded breathing with a respiration belt (Biopac BioNomadix®). Respiration rate (RR) was computed using the Matlab function *findpeaks* after filtering the respiration signal with a bandpass FIR with 0.17 to 0.73 Hz. We identified peaks above at least one robust standard deviation of the filtered respiration time-series [*prctile*(respiration,84.1)-*prctile*(respiration,15.9)]/2, separated by at least 0.8 s and with a minimum width of 0.4 s. The RR was finally computed as the median value of the temporal distance between the identified respiration peaks.

## Behavioral feature description

Behavioral features were split into three categories: movement, trajectory and position; gait; movement burst / immobility.

Movement comprises velocity (vel) and acceleration (acc) captured by the VR headset in the 3 dimensions (x, y and z), resulting in vertical (vert) and horizontal (horz) components, during the 90 seconds, and during the first 20 seconds of each exploration trial. Trajectory features were obtained from [57] and can be consulted in Supplementary Table 1. Position features vary according to the exploration trial (details in Supplementary Table 2). Gait features details in Supplementary Table 3. Movement burst / immobility features were computed by identifying periods respectively with and without more than 3 strides from the motion capture data. For movement burst features see Supplementary Table 4 and for immobility features see Supplementary Table 5. Features that have been collected in the empty room and elevated alley will be followed by (er) and (ea) respectively.

Supplementary Table 1: Trajectory features

| **Variable Name** | **Variable Description** |
|---|---|
| *Eccentricity (er, ea)* | Trajectory eccentricity. How much the trajectory deviates from being circular. Formally, eccentricity is defined as:<br>sqrt(1 - *a*^2/*b*^2)<br>where *a* and *b* are the major and minor axes of a minimum volume ellipsoid that encloses the trajectory points. |
| *Focus (er, ea)* | Trajectory focus. How focused on specific points the trajectory is. Is it broad throughout the entire scenario or focused on specific points. Formally, focus is defined as: |

| | |
|---|---|
| | 1 - a*b/(trajectory_length^2 / (4*pi))<br>where *a* and *b* are the major and minor axes of a minimum volume ellipsoid that encloses the trajectory points. |

Supplementary Table 2: Position features

| Trial | Variable Name | Variable Description |
|---|---|---|
| Empty room (er) | *Wall dist (er)* | Average minimum distance to walls |
| | *Corner dist (er)* | Average minimum distance to corners |
| | *Distance walked (er)* | Total distance walked |
| | *Time on step (er)* | Time in initial red step |
| | *Time in center (er)* | Time in center of room |
| | *Time in edges (er)* | Time in the edges of the room |
| | *Ratio time (er)* | Ratio between time in center and time in edges |
| | *Theta (er)* | Average radius of polar coordinates. |
| | *Ro (er)* | Average angle of coordinates |
| | *Explored (%) (er)* | Percentage of area explored |
| Elevated Alley (ea) | *Time on step (ea)* | Time in initial red step |
| | *Distance walked (ea)* | Total distance walked |
| | *Longitudinal max (ea)* | Maximum longitudinal position |
| | *Latitude unsafe max (ea)* | Maximum lateral position in unsafe area |
| | *Latitude patch 1 max (ea)* | Maximum lateral position in patch 1 |
| | *Latitude patch 2 max (ea)* | Maximum lateral position in patch 2 |
| | *Latitude patch 3 max (ea)* | Maximum lateral position in patch 3 |
| | *Longitudinal unsafe max (ea)* | Maximum longitudinal position in unsafe area |
| | *Longitudinal patch 1 max (ea)* | Maximum longitudinal position in patch 1 |
| | *Longitudinal patch 2 max (ea)* | Maximum longitudinal position in patch 2 |
| | *Longitudinal patch 3 max (ea)* | Maximum longitudinal position in patch 3 |
| | *Theta (ea)* | Average radius of polar coordinates. |
| | *Ro (ea)* | Average angle of coordinates |
| | *Explored (%) (ea)* | Percentage of area explored |
| | *Time unsafe (ea)* | Time spent in unsafe area |
| | *Time safe (ea)* | Time spent in safe area |
| | *Time patch 2 (ea)* | Time spent in patch 2 |
| | *Time patch 3 (ea)* | Time spent in path 3 |

Supplementary Table 3: Gait features

| **Variable Name** | **Variable Description** |
|---|---|
| *GCT_mean (er, ea)* | Average gait cycle time |
| *Stance mean (er, ea)* | Average stance time |
| *Stride length mean (er, ea)* | Average stride length |
| *Stride speed mean (er, ea)* | Average stride speed |
| *Max heel mean (er, ea)* | Maximum heel height |
| *GCT sym (er, ea)* | Symmetry in gait cycle time |
| *Stance sym (er, ea)* | Symmetry in stance time |
| *Stride length sym (er, ea)* | Symmetry in stride length |
| *Stride speed Sym (er, ea)* | Symmetry in stride speed |
| *GCT var (er, ea)* | Standard deviation of gait cycle time |
| *Stance var (er, ea)* | Standard deviation of stance time |
| *Stride length var (er, ea)* | Standard deviation of stride length |
| *Stride speed Var (er, ea)* | Standard deviation of stride speed |
| *Max heel Var (er, ea)* | Standard deviation of heel height |
| *N steps (er, ea)* | Number of steps |

Supplementary Table 4: Movement burst features

| **Variable Name** | **Variable Description** |
|---|---|
| *Burst num (er, ea)* | Number of movement bursts |
| *Burst ons accel (er, ea)* | Average burst onset acceleration (first 1 second) |
| *Burst speed (er, ea)* | Average speed during burst |
| *Burst head scan (er, ea)* | Average head movement during bursts |
| *Burst contrl scan (er, ea)* | Average hand controller movement during bursts |
| *Burst steps (er, ea)* | Average number of steps in burst |
| *Burst leg vel (er, ea)* | Average leg velocity |
| *Burst dur (er, ea)* | Average duration of bursts |
| *Pelv disp (er, ea)* | Amount of vertical pelvis movement during bursts |
| *Burst ecc (er, ea)* | Average eccentricity of trajectory during bursts |
| *Burst focus (er, ea)* | Average focus during bursts |
| *Burst len (er, ea)* | Average distance walked in bursts |
| *Burst ons (er, ea)* | Onset of first burst |

Supplementary Table 5: Immobility features

| **Variable Name** | **Variable Description** |
|---|---|
| *Immmo num (er, ea)* | Number of immobility periods |
| *Immo head scan (er, ea)* | Average head movement during immobility |
| *Immo contrl scan (er, ea)* | Average hand controller movement during immobility |
| *Immo dur (er, ea)* | Average duration of immobility periods |

# Supplementary Results

## Statistics for physiology in exploration scenarios

When exposed to the persistent threat scenario, participants showed autonomic activation (Fig. 2b). Opposingly, when exposed to the virtual empty room (*er*) and the virtual elevated alley (*ea*), participants recovered on average as can be seen in Supplementary Fig. 1.

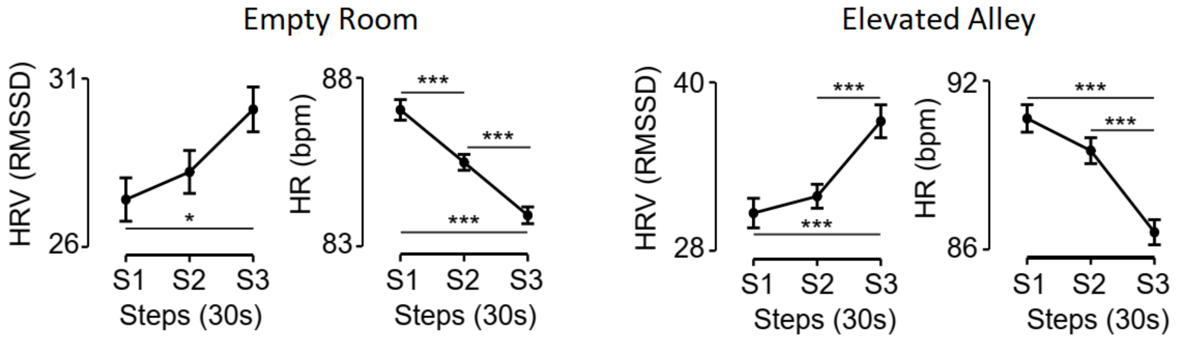


**Supplementary Fig. 1**: Physiological response (HRV ad HR) during the empty room and the elevated alley. N=135 participants examined over 3 consecutive blocks of 30 seconds (S1, S2 and S3). Data are presented as mean values +/- SEM (vertical bars). A repeated measures analysis of variance (rm-ANOVA) was performed for HR and to HRV. Post-hoc tests were performed with two-sided paired t-tests, with *p*-values corrected for multiple comparisons (3 comparisons) with the Holm correction. * *p*-value < 0.05, *** *p*-value < 0.001. Exact *p*-values and statistics are presented in Supplementary Tables 8-11. Source data are provided as a Source Data file.

We analyzed the effects of time (in steps of 30 s) on HR, HRV (with the RMSSD) with a repeated measures analysis of variance (rm-ANOVA). An eventual significant effect of time step suggests that these dependent variables change throughout the exposure to these scenarios and post-hoc analysis will be used to verify the direction of the effect (increase of decrease with time). Post-hoc tests are performed with paired *t*-tests with *p*-values corrected for multiple comparisons (3 comparisons) with the Holm correction. Effect sizes are computed with the Cohen's d for *t*-tests and with the partial Eta squared ($\eta_p^2$) for the effect of time in the rm-ANOVA. Sphericity assumptions are always verified for the rm-ANOVA with the Maulchy's test of sphericity and, if violated, the Greenhouse-Geisser correction to the degrees of freedom is applied.

### Dark maze

For the dark maze (Fig. 2a) a significant effect of time step is present for HR ($F_{2,268}$=16.90, $P$=1.223e -7, $\eta_p^2$=0.112). Post-hoc tests show a significant increase of HR after step S1, as can be seen in Supplementary Table 6.

**Supplementary Table 6**: Post-hoc comparisons between 30 s time steps for the HR, during the dark corridor.

| Post Hoc Comparisons - Steps (30s) | | | | | | |
|---|---|---|---|---|---|---|
| | | Mean Difference | SE | t | Cohen's d | $p_{holm}$ |
| S1 | S2 | -3.363 | 0.689 | -4.877 | 0.420 | 6.004e -6 |
| | S3 | -4.111 | 0.817 | -5.035 | 0.433 | 4.532e -6 |
| S2 | S3 | -0.749 | 0.749 | -1.000 | 0.086 | 0.319 |

A significant effect of time can also be seen in HRV (RMSS) ($F_{2,268}$ =8.74, $P$=2.110e -4, $\eta_p^2$=0.061). Post-hoc tests show a significant decrease in HRV between steps S1 and S3 (Supplementary Table 7).

**Supplementary Table 7**: Post-hoc comparisons between 30 s time steps for the HRV (RMSSD), during the elevated alley.

| **Post Hoc Comparisons - Steps (30s)** | | | | | | |
|---|---|---|---|---|---|---|
| | | **Mean Difference** | **SE** | **t** | **Cohen's d** | **p holm** |
| S1 | S2 | 2.636 | 1.133 | 2.327 | 0.200 | 0.051 |
| | S3 | 4.432 | 1.000 | 4.432 | 0.381 | 5.770e -5 |
| S2 | S3 | 1.796 | 1.063 | 1.689 | 0.145 | 0.093 |

## Empty room

For the empty room (Fig. 1a) a significant effect of time step is present for HR ($F_{1.808,242.226}$=33.626, $P$=1.151e -12, $\eta_p^2$=0.201). Post-hoc tests show a significant decrease of HR with time at each time step, as can be seen in Supplementary Table 8.

**Supplementary Table 8**: Post-hoc comparisons between 30 s time steps for the HR, during the empty room.

| **Post Hoc Comparisons - Steps (30s)** | | | | | | |
|---|---|---|---|---|---|---|
| | | **Mean Difference** | **SE** | **t** | **Cohen's d** | **p holm** |
| S1 | S2 | 1.529 | 0.398 | 3.839 | 0.330 | 1.895e -4 |
| | S3 | 3.080 | 0.411 | 7.490 | 0.645 | 2.497e -11 |
| S2 | S3 | 1.551 | 0.309 | 5.020 | 0.432 | 3.228e -6 |

A significant effect of time can also be seen in HRV (RMSS) ($F_{2,268}$=3.94, $P$=0.021, $\eta_p^2$=0.029). Post-hoc tests show a significant increase in HRV from step S1 to step S3 (Supplementary Table 9).

**Supplementary Table 9**: Post-hoc comparisons between 30 s time steps for the HRV (RMSSD), during the empty room.

| **Post Hoc Comparisons - Steps (30s)** | | | | | | |
|---|---|---|---|---|---|---|
| | | **Mean Difference** | **SE** | **t** | **Cohen's d** | **p holm** |
| S1 | S2 | -0.742 | 0.897 | -0.827 | 0.071 | 0.409 |
| | S3 | -2.502 | 0.925 | -2.706 | 0.233 | 0.023 |
| S2 | S3 | -1.760 | 0.927 | -1.899 | 0.163 | 0.119 |

## Elevated alley

For the elevated alley (Fig. 1b) a significant effect of time step is present for HR ($F_{2,268}$=21.92, $P$=1.523e -9, $\eta_p^2$=0.141). Post-hoc tests show a significant decrease of HR after the time step S2, as can be seen in Supplementary Table 10.

**Supplementary Table 10**: Post-hoc comparisons between 30 s time steps for the HR, during the elevated alley.

| Post Hoc Comparisons - Steps (30s) | | | | | | |
|---|---|---|---|---|---|---|
| | | Mean Difference | SE | t | Cohen's d | p holm |
| S1 | S2 | 0.919 | 0.650 | 1.414 | 0.122 | 0.160 |
| | S3 | 4.035 | 0.670 | 6.026 | 0.519 | 4.593e -8 |
| S2 | S3 | 3.116 | 0.594 | 5.242 | 0.451 | 1.203e -6 |

A significant effect of time can also be seen in HRV (RMSS) ($F_{1.894,237.632}$=11.59, $P$=3.762e -5, $\eta_p^2$=0.080). Similar to HR, post-hoc tests show a significant increase in HRV after step S2 (Supplementary Table 11).

**Supplementary Table 11**: Post-hoc comparisons between 30 s time steps for the HRV (RMSSD), during the elevated alley.

| Post Hoc Comparisons - Steps (30s) | | | | | | |
|---|---|---|---|---|---|---|
| | | Mean Difference | SE | t | Cohen's d | p holm |
| S1 | S2 | -1.396 | 1.231 | -1.134 | 0.098 | 0.259 |
| | S3 | -6.687 | 1.686 | -3.966 | 0.341 | 3.548e -4 |
| S2 | S3 | -5.292 | 1.446 | -3.659 | 0.315 | 7.259e -4 |

## PCA compression for cardiorespiratory variables

Principal component analysis (PCA) was applied to the cardiorespiratory variables (HR, RMSSD, SDNN, HRVTi and RR) in order to obtain a principal component (PC) that represents autonomic influence. Hence, we expected to have a component independent from RR and that would generalize well to the different HRV formulas. Component PC1 (see Supplementary Fig. 2) fulfils these requests.

Correction for outliers was performed before PCA by applying the median absolute deviation (MAD) and replacing the detected outliers with the median of the population. PCA was performed in MATLAB® with the function *PCA*. No rotation was applied.

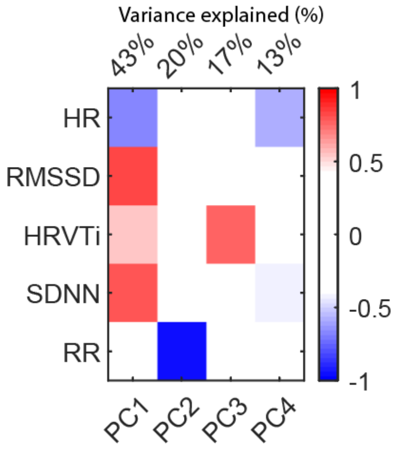


**Supplementary Fig. 2:** Loadings for PCA applied to the cardiorespiratory variables. PC1 explains 43% of the variance in the dataset and loads negatively (blue color) to HR and positively to the HRV variables (mainly RMSSD and SDNN). PC2 loads mainly to the respiration rate negatively and explains 20% of the variance. PC3 loads mainly to the HRVTi formula positively (red color) and explains 17% of the variance. PC4 loads mainly to HR negatively and explains only 13% of the variance. Source data are provided as a Source Data file.

## Model validation on the training dataset

Our model's performance in unseen data from the training dataset was carried out using a 10-fold cross validation that resulted in a significant correlation between the predictions of the 10 folds with the training iHRV (r=0.28, p=0.025, Supplementary Fig. 3). These data indicate that the training set contain more difficult examples than the testing set. Other model evaluation metrics also show a slightly better performance on the test set, but not as pronounced as the correlation values. For the test set (MAE: 1.020, MSE: 1.563, RMSE: 1.251, RMSE/IQR: 0.600) and 10-fold CV train set (MAE: 1.121, MSE: 1.914, RMSE: 1.383, RMSE/IQR: 0.732).

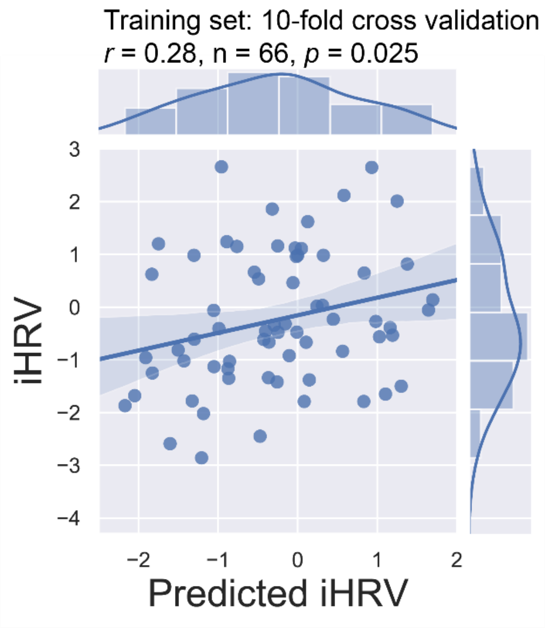


**Supplementary Fig. 3:** Spearman correlation (two-tailed *p*=0.025) between the ***XGBoost*** model's prediction and the integrated HRV index (iHRV) using a 10-fold cross validated performance (i.e., when predictions are made on the unseen data of each one of the 10 folds of cross validation) for the training set. 95% confidence interval for the regression lines drawn using translucent bands. Source data are provided as a Source Data file.

# Selected features

**Supplementary Table 12**: Resulting 18 features after feature selection.

| | | |
|---|---|---|
| **Empty room** | *Focus (er)* | Trajectory focus. |
| | *Corner dist (er)* | Average minimum distance to corners |
| | *Distance walked (er)* | Total distance walked |
| | *Ratio time (er)* | Ratio between time in center and time in edges |
| | *Burst head scan (er)* | Average head movement during bursts |
| | *Burst contrl scan (er)* | Average hand controller movement during bursts |
| | *Immo dur (er)* | Average duration of immobility periods |
| | *GCT var (er)* | Standard deviation of gait cycle time |
| | *Stance var (er)* | Standard deviation of stance time |
| | *Stride speed Var (er)* | Standard deviation of stride speed |
| | *N steps (er)* | Number of steps |
| **Elevated Alley** | *Vertical acceleration (ea)* | Average vertical acceleration during the 90 s. |
| | *Time on step (ea)* | Time in initial red step |
| | *Latitude unsafe max (ea)* | Maximum lateral position in unsafe area |
| | *Longitudinal patch 1 max (ea)* | Maximum longitudinal position in patch 1 |
| | *Time patch 3 (ea)* | Time spent in path 3 |
| | *Horizontal acceleration delta (ea)* | Difference from *er* in average horizontal acceleration during the 90 s in *ea*. |
| | *Vertical acceleration delta (ea)* | Difference from *er* in average vertical acceleration during the 90 s in *ea*. |

# XGBoost algorithm

After Bayesian optimization, the resulting hyperparamteres for the model were:

(n_estimators = 8, colsample_bytree=0.84, learning_rate = 0.546, max_depth = 6, min_child_weight = 5, subsample = 0.724, gamma = 0.427, reg_lambda = 0.59, reg_alpha = 0.67)

The resulting decision trees for the 8 estimators can be in Supplementary Figs 4-11:

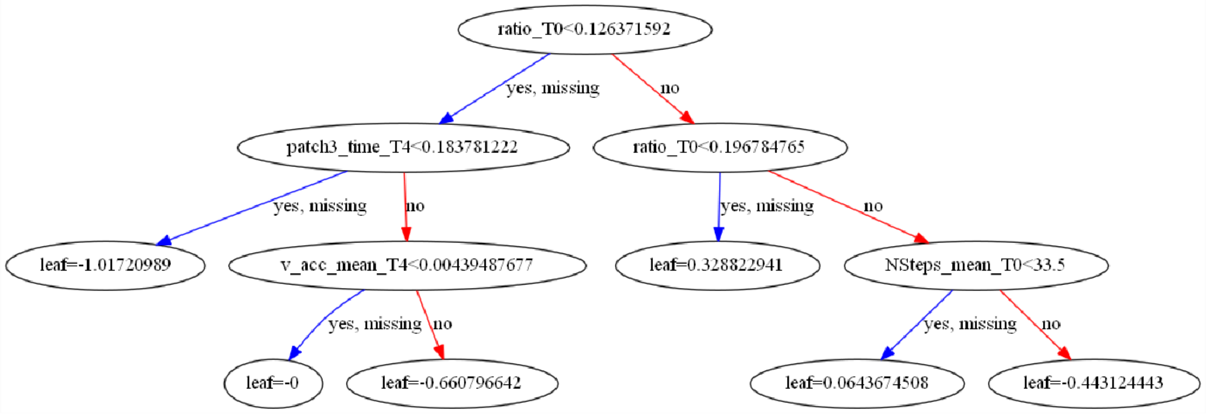


**Supplementary Fig. 4**: Decision tree for estimator 1.

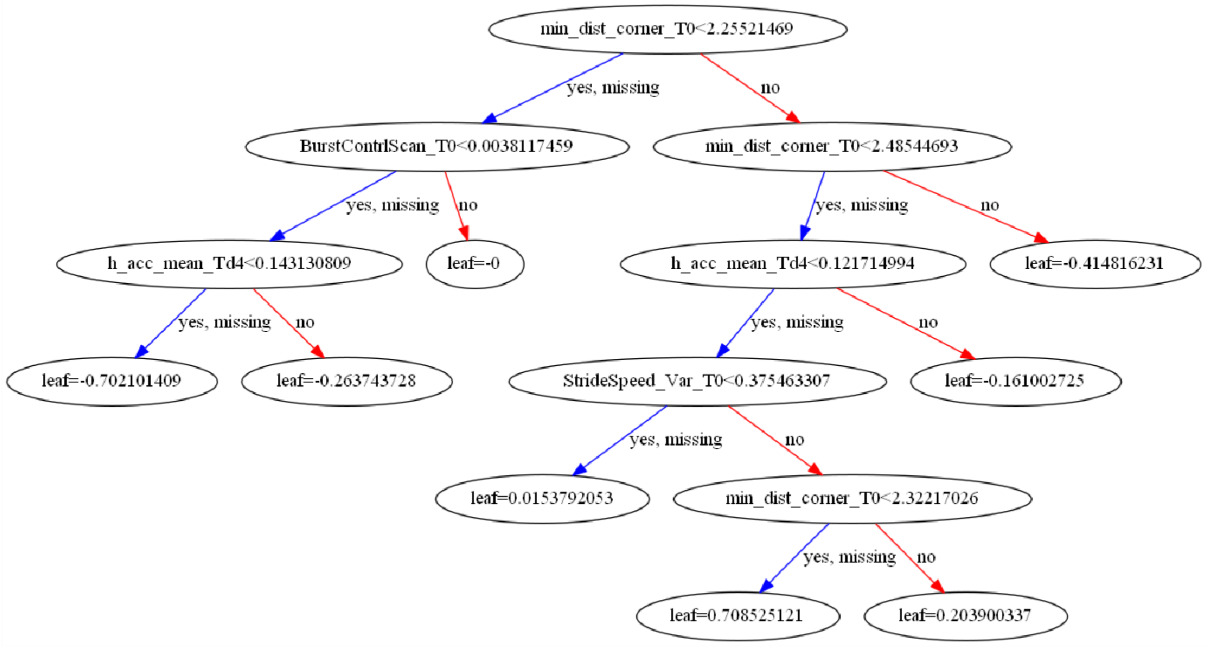


**Supplementary Fig. 5**: Decision tree for estimator 2.

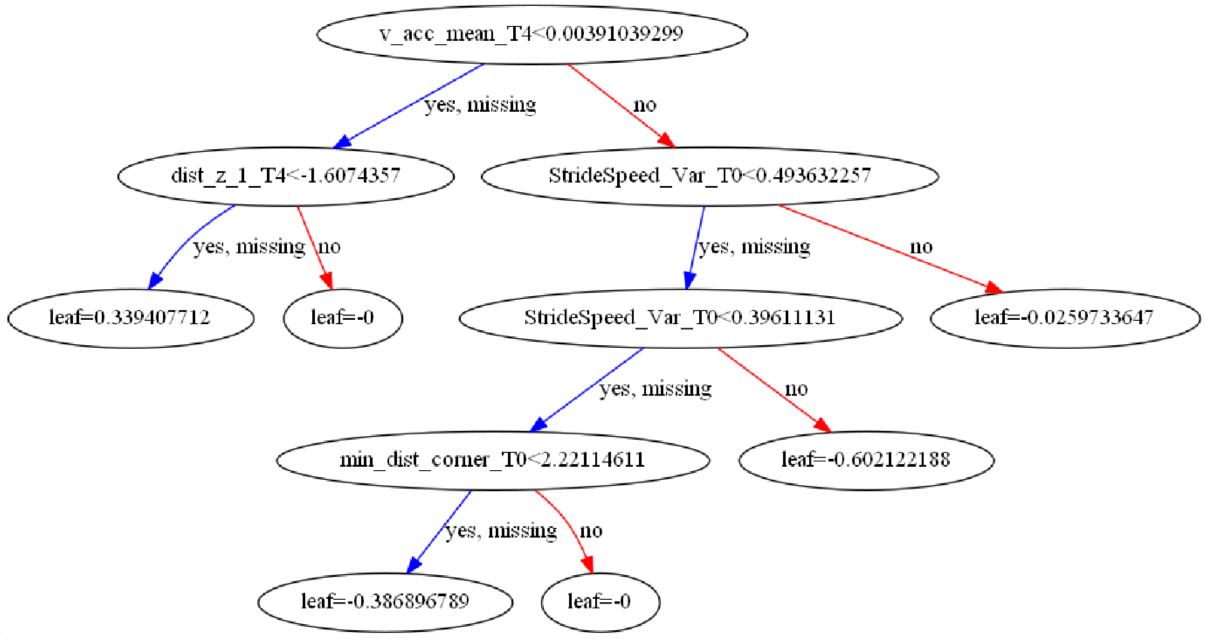


**Supplementary Fig. 6**: Decision tree for estimator 3.

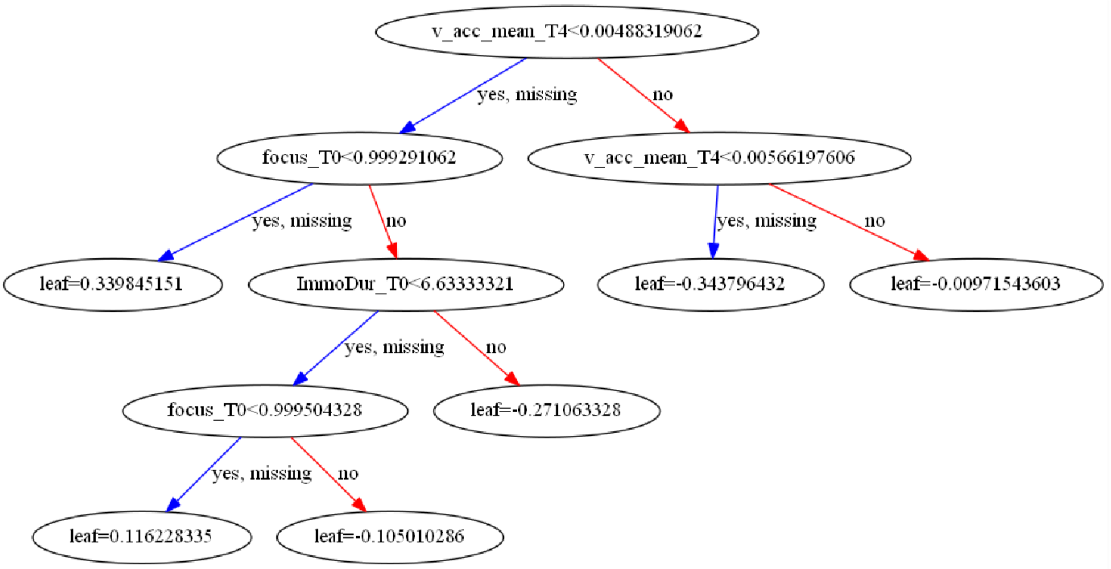


**Supplementary Fig. 7**: Decision tree for estimator 4.

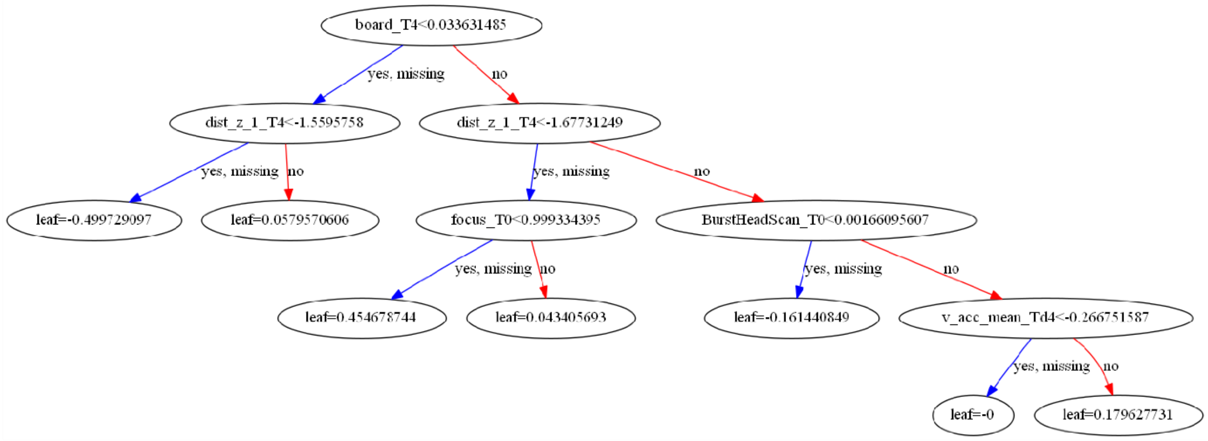


**Supplementary Fig. 8**: Decision tree for estimator 5.

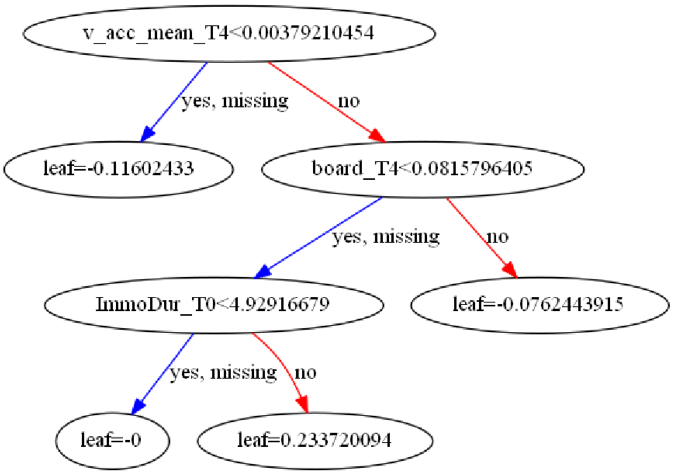


**Supplementary Fig. 9**: Decision tree for estimator 6.

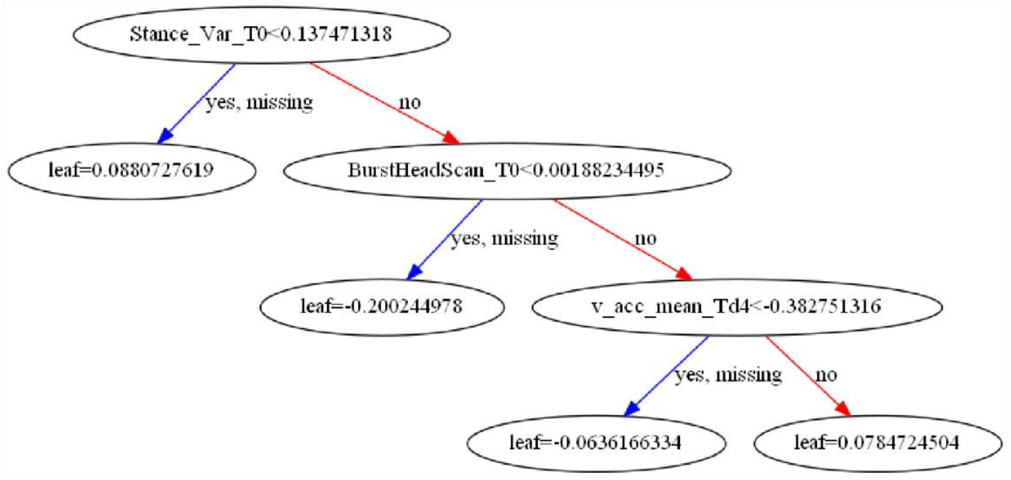


**Supplementary Fig. 10**: Decision tree for estimator 7.

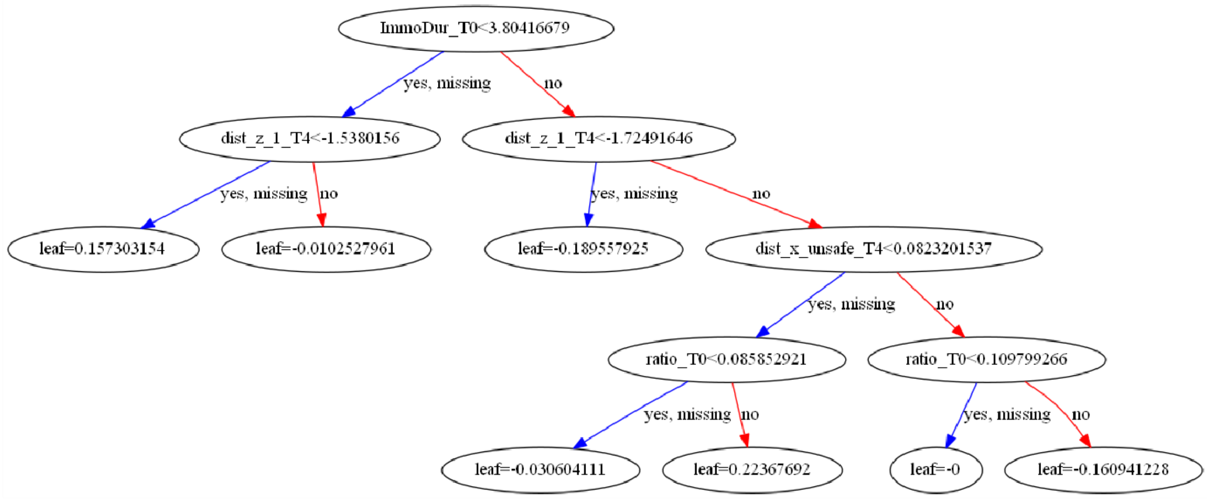


**Supplementary Fig. 11**: Decision tree for estimator 8.

Finally, the model's final decision based on the 8 estimators results in the scores presented in Supplementary Fig. 12 compared to the actual scores. The contribution of the behavioral features is informed by the SHAP values, which represent how the features, for each individual, influence the model's output (negatively or positively).

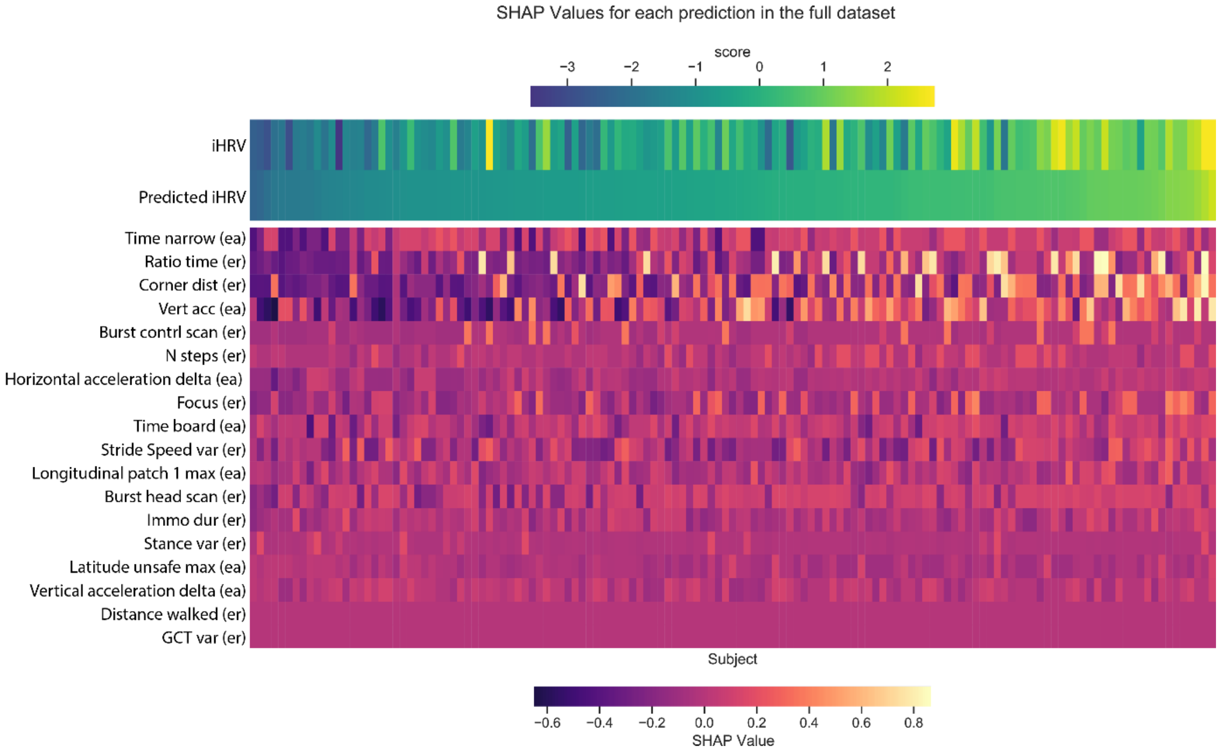


**Supplementary Fig. 12**: True PC1 scores versus model's prediction (upper *viridis* color scale panel). SHAP values (lower *magma* color panel). Source data are provided as a Source Data file.

## Statistics for physiology in Fig. 4b

We analyzed the effects of the stress task (in four steps of 2.5 minutes during the stress task and one 2.5 minutes baseline) on HR and HRV (with the RMSSD) with a repeated measures analysis of variance (rm-ANOVA) in the generalization sample. Due to excessive movement artifact in the 2.5 minutes steps ECG, a total 8 subjects were removed for this analysis. An eventual significant effect of time step suggests that these dependent variables change throughout the exposure to the stress task in comparison to the baseline or within the stress test. Post-hoc tests will be used upon a significant effect of time to verify the direction and location of the effects (increase of decrease with time with relation to the baseline or within the stress test). Post-hoc tests are performed with paired *t*-tests with *p*-values corrected for multiple comparisons (10 comparisons) with the Holm correction. Effect sizes are computed with the Cohen's d for *t*-tests and with the partial Eta squared ($\eta_p^2$) for the effect of time in the rm-ANOVA. Sphericity assumptions are always verified for the rm-ANOVA analyses with the Maulchy's test of sphericity and, if violated, the Greenhouse-Geisser correction to the degrees of freedom is applied.

## Physiology analysis

A significant effect of time step is present for HR ($F_{2.701,264}$=41.14, $P$<0.001, $\eta_p^2$=0.296) as can be seen in Supplementary Table 13 post-hoc tests show a significant large increase of HR compared to the baseline step BL, and a consistent gradual increase after step S2.

**Supplementary Table 13**: Post-hoc comparisons between baseline (BL) and stress (S1-4) time steps for the HR.

| **Post Hoc Comparisons - Stress** | | | | | | |
|---|---|---|---|---|---|---|
| | | **Mean Difference** | **SE** | **t** | **Cohen's d** | **$p_{holm}$** |
| BL | S1 | -5.266 | 0.647 | -8.137 | 0.818 | 1.034e -11 |
| | S2 | -5.156 | 0.728 | -7.080 | 0.712 | 1.535e -9 |
| | S3 | -6.290 | 0.704 | -8.932 | 0.898 | 2.263e -13 |
| | S4 | -7.408 | 0.812 | -9.125 | 0.917 | 9.609e -14 |
| S1 | S2 | 0.110 | 0.575 | 0.192 | 0.019 | 0.848 |
| | S3 | -1.024 | 0.626 | -1.637 | 0.165 | 0.210 |
| | S4 | -2.141 | 0.719 | -2.977 | 0.299 | 0.015 |
| S2 | S3 | -1.134 | 0.430 | -2.638 | 0.265 | 0.029 |
| | S4 | -2.252 | 0.534 | -4.215 | 0.424 | 3.338e -4 |
| S3 | S4 | -1.117 | 0.353 | -3.161 | 0.318 | 0.010 |

A significant effect of time step is present for HRV (RMSSD) ($F_{2.914,285.546}$=12.96, $P$<0.001, $\eta_p^2$=0.117). As can be seen in Supplementary Table 14 post-hoc tests show a significant decrease of HRV in steps S3 and S4 when compared to the baseline step BL and to steps S1 and S2.

**Supplementary Table 14**: Post-hoc comparisons between baseline (BL) and stress (S1-4) time steps for the HRV (RMSSD).

| **Post Hoc Comparisons - Stress** | | | | | | |
|---|---|---|---|---|---|---|
| | | **Mean Difference** | **SE** | **t** | **Cohen's d** | **$p_{holm}$** |
| BL | S1 | 1.781 | 1.022 | 1.743 | 0.175 | 0.253 |
| | S2 | 2.679 | 1.066 | 2.513 | 0.253 | 0.054 |
| | S3 | 5.243 | 1.021 | 5.136 | 0.516 | 1.425e -5 |
| | S4 | 5.167 | 1.084 | 4.767 | 0.479 | 5.855e -5 |
| S1 | S2 | 0.898 | 0.884 | 1.015 | 0.102 | 0.625 |
| | S3 | 3.463 | 0.831 | 4.167 | 0.419 | 5.333e -4 |
| | S4 | 3.386 | 0.908 | 3.728 | 0.375 | 0.002 |
| S2 | S3 | 2.565 | 0.669 | 3.835 | 0.385 | 0.002 |
| | S4 | 2.488 | 0.695 | 3.579 | 0.360 | 0.003 |
| S3 | S4 | -0.077 | 0.463 | -0.166 | 0.017 | 0.869 |

## Stress test

Stress was assessed using an adaptation of the Montreal Imaging Stress Task (MIST) [58] to virtual reality, itself derived from the widely-validated Trier Mental Challenge Test [59]. Designed to investigate stress in a constrained functional imaging environment, the MIST experimental condition consists of a succession of mental arithmetic questions which are manipulated to induce a high failure rate in each participant. Feedback is provided after each response ("correct" or "incorrect"), and two performance measures are displayed at all times: individual average performance and average performance of all participants. The latter, rigged to be higher than individual performance, adds a psychosocial evaluative threat component to the task, which has been demonstrated to help induce stress [58,60,61]. Psychosocial stress in the MIST is supplemented by intermittent reminders during the task that performance needs to be improved, as well as prior instructions stating that individual performance is being monitored by experimenters.

In our adaptation, all components were implemented in a virtual reality setting, consisting of an empty room with tiled flooring in which the participant could navigate by walking. Mental arithmetic questions appeared in the heads-up display and could be answered by pressing on two controller buttons ("True" or "False"). Participant's performance was compared to average performance from other participants and, similarly to the MIST, the level of challenge was titrated for performance to be below this average. A wrong response would result in a tile exploding, through were the participant could fall. Eventually the participant would fall due to a navigation mistake or to the lack of tiles to stand on. A fast-paced classical music played in the background. Participants were free to move around the room at will and were requested to avoid falling in holes and to perform as well as possible. Three intermittent reminders were given during the task informing the participant that performance needs to be improved. In the second reminder, the experiment restarts since performance was presumably too low. To further increase the sense of uncontrollability [61], there was a 5% chance of a correct response to be taken as false.

At the end of the experiment, participants were debriefed on the purpose of the experiment, that performance was artificially lowered to be below a fake average and notified that mental arithmetic ability was not being recorded.

In addition, a second block was added in which participants were exposed to a 'control' and relaxing VR scenario, in which physiological measures were taken as baseline.

## Sustained anticipatory anxiety paradigm: Fear conditioning test

Several days prior to the tasks in VR, participants were invited to participate in a fear conditioning experiment, based on a classical differential delayed fear conditioning paradigm [62], at our laboratory. We considered the habituation phase of this test as a sustained anticipatory anxiety paradigm since participants were informed that shocks would be administered but at some point, but during habituation shocks are never delivered. They sat 50 cm in front of a 24 inch computer screen, resting their chin on the the EyeLink 1000 chinrest, which was used to track the gaze and pupil dilation. Pulse and skin conductance were measured from the participant's left had, which was resting on a cushion on the table, with a Biopac MP150 device. Electric shocks were delivered by an electric shocker from Psychlab Contact Precision Instruments plugged into the computer's USB port. Shocks lasted 0.5 s and their intensity ranged from 2 mA to 5 mA, which was adapted for each participant following the shock workup suggested in [63]: four shock samples are administered, starting at 2 mA up to a

maximum of 5 mA. Participants are asked to rated each shock on a scale from 1 (barely felt) to 5 (very unpleasant / uncomfortable). The shock increases in steps of 1 mA until the rating of 4 is reached. If the rating of 4 is reached before the fourth shock, additional shocks of the current intensity are given to reach a total of four shocks. If the rating of 4 is not reached after four shocks, the maximum intensity of 5 mA is used. Fear conditioning consisted of an initial anticipation block where participants are exposed to the two neutral stimuli (and no shocks are delivered (even though participants are expecting shocks to be delivered), two fear acquisition blocks where one of the neutral stimuli is paired with the electric shock (CS+) 50% of the time and the other stimulus is left unpaired (CS-), and an extinction block where the CS+ is no longer paired with the electric shock. During anticipation, no shocks were delivered and participants are only exposed to the neutral images (CS+ and CS-) during 5 s, separated by a fixation cross with a jittered interval from 4.5 to 7.5 s. Both the CS+ and CS- images are presented in a pseudorandom order 6 times each. During each acquisition block shocks are delivered (lasting 0.5 s) at the end of the CS+ stimulus (4.5 s after CS+ presentation) 50% of the times the CS+ is presented. The fixation cross separates stimuli presentation with a jittered interval from 7.5 to 12.5 s. Both the CS+ and CS- images are presented in a pseudorandom order 16 times each, 8 shocks are delivered. At the beginning of the experiment, participants were informed by written instructions that images will be presented and that socks will be delivered at certain moments. No information was given regarding the contingency.

## Conditioning

Conditioning is successfully achieved as can be seen in Supplementary Fig. 13 from the effect of fear conditioning block on the difference in skin conductance response between the CS+ and CS- (delta EDA AUC; $F_{1.467,151.132}$=6.55, $P$=0.005, $\eta_p^2$=0.060) in the generalization sample. Due to missing EDA and pule data, 3 subjects were removed for this analysis. Both acquisition blocks are significantly different (see post-hoc tests in Supplementary Table 15), with larger skin conductance differences ($M_{acq1}$ = 10.39 $SD_{acq1}$=14.92; $M_{acq2}$ = 8.86 $SD_{acq2}$=32.69), than the anticipation block ($M_{hab}$ = 0.23 $SD_{hab}$=13.65).

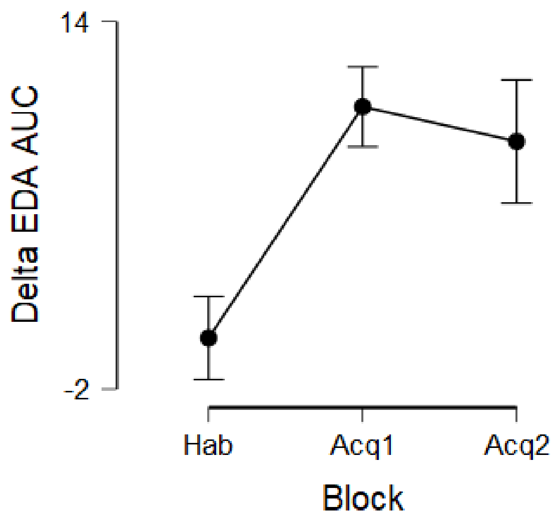


**Supplementary Fig. 13**: Difference in total skin conductance response (area under the curve – AUC) between CS+ and CS- during the three fear conditioning blocks. A positive difference is expected if conditioning is successful since the CS+ should elicit a response while the CS- shouldn't. N=104 participants examined over 3

consecutive experimental blocks (Hab, Acq1 and Acq2). Data are presented as mean values +/- SEM (vertical bars). Source data are provided as a Source Data file.

**Supplementary Table 15**: Post-hoc comparisons between habituation (Hab) and acquisition (Acq1 and Acq2) blocks for the difference in skin conductance responses between CS+ and CS-.

| **Post Hoc Comparisons - Block** | | | | | | |
|---|---|---|---|---|---|---|
| | | **Mean Difference** | **SE** | **t** | **Cohen's d** | **p holm** |
| Hab | Acq1 | -10.157 | 1.910 | -5.319 | 0.522 | 1.829e -6 |
| | Acq2 | -8.631 | 3.488 | -2.474 | 0.243 | 0.030 |
| Acq1 | Acq2 | 1.527 | 3.412 | 0.447 | 0.044 | 0.656 |

## Possible higher anxiety during anticipation

Due to the expectation of a shock already during anticipation (which was never delivered in this block), and for this block to be the first, hence more novelty, we expect participants to be in a higher state of anxiety here. This is suggested by a larger skin conductance response to CS- stimuli during habituation than acquisition blocks (Supplementary Fig. 14; $F_{1.719.701,177.019}$=8.79, $P$<0.001, $\eta_p^2$=0.079). The skin conductance response in the anticipation block is significantly higher ($M_{hab}$ = 1123.29 $SD_{hab}$=9.71; see post-hoc tests in Supplementary Table 16) than in both acquisition blocks ($M_{acq1}$ = 1118.93 $SD_{acq1}$=8.56; $M_{acq2}$ = 1119.07 $SD_{acq2}$=9.00).

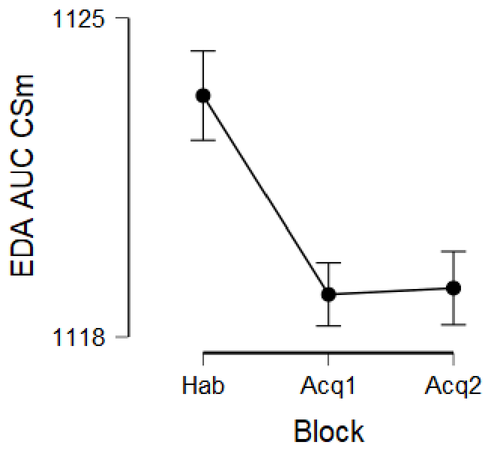


**Supplementary Fig. 14**: total skin conductance response (area under the curve – AUC) for CS- stimuli during the three fear conditioning blocks. N=104 participants examined over 3 consecutive experimental blocks (Hab, Acq1 and Acq2). Data are presented as mean values +/- SEM (vertical bars). Source data are provided as a Source Data file.

**Supplementary Table 16**: Post-hoc comparisons between habituation (Hab) and acquisition (Acq1 and Acq2) blocks for the skin conductance responses to CS- stimuli.

| **Post Hoc Comparisons - Block** | | | | | | |
|---|---|---|---|---|---|---|
| | | **Mean Difference** | **SE** | **t** | **Cohen's d** | **p holm** |
| Hab | Acq1 | 4.358 | 1.221 | 3.568 | 0.350 | 0.002 |
| | Acq2 | 4.218 | 1.352 | 3.119 | 0.306 | 0.005 |
| Acq1 | Acq2 | -0.139 | 0.930 | -0.150 | 0.015 | 0.881 |

## Supplementary References


1. Crick, S. J. *et al.* Innervation of the human cardiac conduction system: A quantitative immunohistochemical and histochemical study. *Circulation* **89**, 1697–1708 (1994).

2. Burkholder, T. *et al.* Gross and microscopic anatomy of the vagal innervation of the rat heart. *Anat. Rec.* **232**, 444–452 (1992).

3. Levy, M. . & Warner, M. . Parasympathetic effects of cardiac function. in *Neurocardiology* (eds. Armour, J. & Ardell, J.) 53–76 (Oxford University Press, 1994).

4. Randall, W. C. Efferent sympathetic innervation of the heart. in *Neurocardiology* (eds. Armour, J. & Ardell, J.) 77–94 (Oxford University Press, 1994).

5. Levy, M. N. Cardiac sympathetic-parasympathetic interactions. *Fed. Proc.* **43**, 2598–2602 (1984).

6. Moreno, A. *et al.* Sudden heart rate reduction upon optogenetic release of acetylcholine from cardiac parasympathetic neurons in perfused hearts. *Front. Physiol.* **10**, 1–11 (2019).

7. Peňáz, J. Frequency response of the cardiac chronotropic action of the vagus in the rabbit. *Arch. Physiol. Biochem.* **70**, 636–650 (1962).

8. Berger, R. D., Saul, J. P. & Cohen, R. J. Transfer function analysis of autonomic regulation. I. Canine atrial rate response. *Am. J. Physiol. - Hear. Circ. Physiol.* **256**, (1989).

9. Spear, J. F., Kronhaus, K. D., Moore, E. N. & Kline, R. P. The effect of brief vagal stimulation on the isolated rabbit sinus node. *Circ. Res.* **44**, 75–88 (1979).

10. Venables, P. H. Autonomic Activity. *Ann. N. Y. Acad. Sci.* **620**, 191–207 (1991).

11. Ramírez, E., Ortega, A. R. & Reyes Del Paso, G. A. Anxiety, attention, and decision making: The moderating role of heart rate variability. *Int. J. Psychophysiol.* **98**, 490–496 (2015).

12. Friedman, B. H. An autonomic flexibility-neurovisceral integration model of anxiety and cardiac vagal tone. *Biol. Psychol.* **74**, 185–199 (2007).

13. Chalmers, J. A., Heathers, J. A. J., Abbott, M. J., Kemp, A. H. & Quintana, D. S. Worry is associated with robust reductions in heart rate variability: A transdiagnostic study of anxiety psychopathology. *BMC Psychol.* **4**, 1–9 (2016).

14. Kok, B. E. & Fredrickson, B. L. Upward spirals of the heart: Autonomic flexibility, as indexed by vagal tone, reciprocally and prospectively predicts positive emotions and social connectedness. *Biol. Psychol.* **85**, 432–436 (2010).

15. Quintana, D. S., Guastella, A. J., Outhred, T., Hickie, I. B. & Kemp, A. H. Heart rate variability is associated with emotion recognition: Direct evidence for a relationship between the autonomic nervous system and social cognition. *Int. J. Psychophysiol.* **86**, 168–172 (2012).

16. Brosschot, J. F., Verkuil, B. & Thayer, J. F. The default response to uncertainty and the importance of perceived safety in anxiety and stress: An evolution-theoretical perspective. *J. Anxiety Disord.* **41**, 22–34 (2015).

17. Thayer, J. F. & Lane, R. D. The role of vagal function in the risk for cardiovascular disease and mortality. *Biol. Psychol.* **74**, 224–242 (2007).

18. Thayer, J. F., Yamamoto, S. S. & Brosschot, J. F. The relationship of autonomic imbalance, heart rate variability and cardiovascular disease risk factors. *Int. J. Cardiol.* **141**, 122–131 (2010).

19. Thayer, J. F., Åhs, F., Fredrikson, M., Sollers, J. J. & Wager, T. D. A meta-analysis of heart rate variability and neuroimaging studies: Implications for heart rate variability as a marker of stress and health. *Neuroscience and Biobehavioral Reviews* **36**, 747–756 (2012).

20. Kemp, A. H. & Quintana, D. S. The relationship between mental and physical health: Insights from the study of heart rate variability. *International Journal of Psychophysiology* **89**, 288–296 (2013).

21. Beauchaine, T. P. & Thayer, J. F. Heart rate variability as a transdiagnostic biomarker of psychopathology. *Int. J. Psychophysiol.* **98**, 338–350 (2015).

22. Ottaviani, C. *et al.* Physiological concomitants of perseverative cognition: A systematic review and meta-analysis. *Psychol. Bull.* **142**, 231–259 (2016).

23. Chalmers, J. A., Quintana, D. S., Abbott, M. J. A. & Kemp, A. H. Anxiety disorders are associated with reduced heart rate variability: A meta-analysis. *Front. Psychiatry* **5**, 1–11 (2014).

24. Appelhans, B. M. & Luecken, L. J. Heart rate variability as an index of regulated emotional responding. *Rev. Gen. Psychol.* **10**, 229–240 (2006).

25. Schäfer, A. & Vagedes, J. How accurate is pulse rate variability as an estimate of heart rate variability?: A review on studies comparing photoplethysmographic technology with an electrocardiogram. *Int. J. Cardiol.* **166**, 15–29 (2013).

26. Pinheiro, N. *et al.* Can PPG be used for HRV analysis? *Proc. Annu. Int. Conf. IEEE Eng. Med. Biol. Soc. EMBS* **2016-Octob**, 2945–2949 (2016).

27. Vasilev, C. A., Crowell, S. E., Beauchaine, T. P., Mead, H. K. & Gatzke-Kopp, L. M. Correspondence between physiological and self-report measures of emotion dysregulation: A longitudinal investigation of youth with and without psychopathology. *J. Child Psychol. Psychiatry Allied Discip.* **50**, 1357–1364 (2009).

28. Beauchaine, T. P. Respiratory sinus arrhythmia: A transdiagnostic biomarker of emotion dysregulation and psychopathology. *Curr. Opin. Psychol.* **3**, 43–47 (2015).

29. Hastings, P. D. *et al.* Applying the polyvagal theory to children's emotion regulation: Social context, socialization, and adjustment. *Biol. Psychol.* **79**, 299–306 (2008).

30. Kemp, A. H. *et al.* Effects of depression, anxiety, comorbidity, and antidepressants on resting-state heart rate and its variability: An ELSA-Brasil cohort baseline study. *Am. J. Psychiatry* **171**, 1328–1334 (2014).

31. Åhs, F., Sollers, J. J., Furmark, T., Fredrikson, M. & Thayer, J. F. High-frequency heart rate variability and cortico-striatal activity in men and women with social phobia. *Neuroimage* **47**, 815–820 (2009).

32. De Wied, M., Van Boxtel, A., Matthys, W. & Meeus, W. Verbal, facial and autonomic responses to empathy-eliciting film clips by disruptive male adolescents with high versus low callous-unemotional traits. *J. Abnorm. Child Psychol.* **40**, 211–223 (2012).

33. Beauchaine, T. P., Gatzke-Kopp, L. & Mead, H. K. Polyvagal Theory and developmental psychopathology: Emotion dysregulation and conduct problems from preschool to adolescence. *Biol. Psychol.* **74**, 174–184 (2007).

34. Rottenberg, J., Salomon, K., Gross, J. J. & Gotlib, I. H. Vagal withdrawal to a sad film predicts subsequent recovery from depression. *Psychophysiology* **42**, 277–281 (2005).

35. Rottenberg, J. Cardiac vagal control in depression: A critical analysis. *Biol. Psychol.* **74**, 200–211 (2007).

36. J., R., F.H., W., J.J., G. & I.H., G. Respiratory sinus arrhythmia as a predictor of outcome in major depressive disorder. *J. Affect. Disord.* **71**, 265–272 (2002).

37. Sloan, R. P. *et al.* Cardiac autonomic control and hostility in healthy subjects. *Am. J. Cardiol.* **74**, 298–300 (1994).

38. Hansen, A. L., Johnsen, B. H., Thornton, D., Waage, L. & Thayer, J. F. Facets of psychopathy, heart rate variability and cognitive function. *J. Pers. Disord.* **21**, 568–582 (2007).

39. Porges, S. W. The polyvagal perspective. *Biol. Psychol.* **74**, 116–143 (2007).

40. Stroud, L. R. *et al.* Stress response and the adolescent transition: Performance versus peer rejection stressors. *Dev. Psychopathol.* **21**, 47–68 (2009).

41. Riva, G. *et al.* Affective interactions using virtual reality: the link between presence and emotions. *Cyberpsychol. Behav.* **10**, 45–56 (2007).

42. Macedonio, M. F., Parsons, T. D., Digiuseppe, R. A., Weiderhold, B. A. & Rizzo, A. A. Immersiveness and Physiological Arousal within Panoramic Video-Based Virtual Reality. *CyberPsychology Behav.* **10**, 508–515 (2007).

43. Bullinger, A. H. *et al.* Stimulation of cortisol during mental task performance in a provocative virtual environment. *Appl. Psychophysiol. Biofeedback* **30**, 205–216 (2005).

44. Bergström, I., Kilteni, K. & Slater, M. First-Person Perspective Virtual Body Posture Influences Stress: A Virtual Reality Body Ownership Study. *PLoS One* **11**, 1–21 (2016).

45. Cornwell, B. R., Johnson, L., Berardi, L. & Grillon, C. Anticipation of public speaking in virtual reality reveals a relationship between trait social anxiety and startle reactivity. *Biol. Psychiatry* **59**, 664–666 (2006).

46. Ziemssen, T., Gasch, J. & Ruediger, H. Influence of ECG sampling frequency on spectral analysis of RR intervals and baroreflex sensitivity using the EUROBAVAR data set. *J. Clin. Monit. Comput.* **22**, 159–168 (2008).

47. Task Force of the European Society of Cardiology and the North American Society of Pacing and Electrophysiology. Heart rate variability: Standards of measurement, physiological interpretation, and clinical use. *Circulation* **93**, 1043–1065 (1996).

48. Pan, J. & Tompkins, W. J. Real-Time Qrs Detection Algorithm. *IEEE Trans. Biomed. Eng.* **BME-32**, 230–236 (1985).

49. Castaldo, R., Montesinos, L., Melillo, P., James, C. & Pecchia, L. Ultra-short term HRV features as surrogates of short term HRV: A case study on mental stress detection in real life. *BMC Med. Inform. Decis. Mak.* **19**, 1–13 (2019).

50. Salahuddin, L., Cho, J., Jeong, M. G. & Kim, D. Ultra Short Term Analysis of Heart Rate Variability for Monitoring Mental Stress in Mobile Settings. *2007 29th Annu. Int. Conf. IEEE Eng. Med. Biol. Soc.* 4656–4659 (2007).

51. Pecchia, L., Castaldo, R., Montesinos, L. & Melillo, P. Are ultra-short heart rate variability features good surrogates of short-term ones? State-of-the-art review and recommendations. *Healthc. Technol. Lett.* **5**, 94–100 (2018).

52. Li, K., Rüdiger, H. & Ziemssen, T. Spectral Analysis of Heart Rate Variability : Time Window Matters. **10**, 1–12 (2019).

53. Berntson, G. G. *et al.* Heart rate variability: Origins, methods and interpretive caveats. *Psychophysiology, 34* 623–648 (1997).

54. Penttilä, J. *et al.* Time domain, geometrical and frequency domain analysis of cardiac vagal outflow: Effects of various respiratory patterns. *Clin. Physiol.* **21**, 365–376 (2001).

55. Hill, L. B. K., Siebenbrock, A., Sollers, J. J. & Thayer, J. F. Are all measures created equal? Heart rate variability and respiration. *46th Annu. Rocky Mt. Bioeng. Symp. 46th Int. ISA Biomed. Sci. Instrum. Symp. 2009* **476**, 71–76 (2009).

56. Acharya, U. R., Joseph, K. P., Kannathal, N., Lim, C. M. & Suri, J. S. Heart rate variability: A review. *Med. Biol. Eng. Comput.* **44**, 1031–1051 (2006).

57. Vouros, A. *et al.* A generalised framework for detailed classification of swimming paths inside the Morris Water Maze. *Sci. Rep.* **8**, 1–15 (2018).

58. Dedovic, K. *et al.* The Montreal Imaging Stress Task: Using functional imaging to investigate the effects of perceiving and processing psychosocial stress in the human brain. *J. Psychiatry Neurosci.* **30**, 319–325 (2005).

59. Kirschbaum, C., Pirke, K. M. & Hellhammer, D. H. The 'Trier Social Stress Test'--a tool for investigating psychobiological stress responses in a laboratory setting. *Neuropsychobiology* **28**, 76–81 (1993).

60. Dickerson, S. S. & Kemeny, M. E. Acute stressors and cortisol responses: a theoretical integration and synthesis of laboratory research. *Psychol. Bull.* **130**, 355–391 (2004).

61. Allen, A. P. *et al.* The Trier Social Stress Test: Principles and practice. *Neurobiol. Stress* **6**, 113–126 (2017).

62. Veit, R. *et al.* Deficient fear conditioning in psychopathy as a function of interpersonal and affective disturbances. *Front. Hum. Neurosci.* **7**, 1–12 (2013).

63. Schmitz, A. & Grillon, C. Assessing fear and anxiety in humans using the threat of predictable and unpredictable aversive events (the NPU-threat test). *Nat. Protoc.* **7**, 527–32 (2012).
