# Supplementary material for: Locomotion in virtual environments predicts cardiovascular responsiveness to subsequent stressful challenges
Source: Nat Commun. 2020 Nov 19;11:5904. doi: 10.1038/s41467-020-19736-3 (PMC7677550; doi:10.1038/s41467-020-19736-3)
Supplement: Supplementary file 3 — Reporting Summary [file 41467_2020_19736_MOESM3_ESM.pdf]

nature research

Corresponding author(s): Carmen Sandi
João Rodrigues

Last updated by author(s): Sep 25, 2020

Reporting Summary

Nature Research wishes to improve the reproducibility of the work that we publish. This form provides structure for consistency and transparency in reporting. For further information on Nature Research policies, see our Editorial Policies and the Editorial Policy Checklist.

Please do not complete any field with "not applicable" or n/a. Refer to the help text for what text to use if an item is not relevant to your study.
For final submission: please carefully check your responses for accuracy; you will not be able to make changes later.

Statistics

For all statistical analyses, confirm that the following items are present in the figure legend, table legend, main text, or Methods section.

n/a	Confirmed	
☐	☒	The exact sample size (*n*) for each experimental group/condition, given as a discrete number and unit of measurement
☐	☒	A statement on whether measurements were taken from distinct samples or whether the same sample was measured repeatedly
☐	☒	The statistical test(s) used AND whether they are one- or two-sided<br>*Only common tests should be described solely by name; describe more complex techniques in the Methods section.*
☒	☐	A description of all covariates tested
☐	☒	A description of any assumptions or corrections, such as tests of normality and adjustment for multiple comparisons
☐	☒	A full description of the statistical parameters including central tendency (e.g. means) or other basic estimates (e.g. regression coefficient) AND variation (e.g. standard deviation) or associated estimates of uncertainty (e.g. confidence intervals)
☐	☒	For null hypothesis testing, the test statistic (e.g. *F*, *t*, *r*) with confidence intervals, effect sizes, degrees of freedom and *P* value noted<br>*Give P values as exact values whenever suitable.*
☒	☐	For Bayesian analysis, information on the choice of priors and Markov chain Monte Carlo settings
☒	☐	For hierarchical and complex designs, identification of the appropriate level for tests and full reporting of outcomes
☐	☒	Estimates of effect sizes (e.g. Cohen's *d*, Pearson's *r*), indicating how they were calculated

Our web collection on statistics for biologists contains articles on many of the points above.

Software and code

Policy information about availability of computer code

Data collection	Physiological data was recorded with the AcqKnowledge Data Acquisition and Analysis Software 5.0 from Biopac. Motion capture data was recorded with the MVN XSENS Awinda system and the MVN Studio Animate Pro 2019.
Data analysis	Heart rate calculation was done in AcqKnowledge Data Acquisition and Analysis Software 5.0 from Biopac. Heart rate variability was done in Matlab 2018b. Gait analysis was performed in Matlab 2018b. Feature selection was performed in Matlab 2018b. Machine learning using the Python packages xgboost v0.82 , pandas v1.05, numpy v1.18.5, hyperopt v0.1.2, datacleaner v0.1.5, shap v0.30 was performed in the Python 3.7.1 environment, with JupyterLab 0.35.3. Correlations and multiple comparison correction were performed in the R environment using R version 3.5.3 and JASP 0.11.1.0.

For manuscripts utilizing custom algorithms or software that are central to the research but not yet described in published literature, software must be made available to editors and reviewers. We strongly encourage code deposition in a community repository (e.g. GitHub). See the Nature Research guidelines for submitting code & software for further information.

Data

Policy information about availability of data

All manuscripts must include a data availability statement. This statement should provide the following information, where applicable:

- Accession codes, unique identifiers, or web links for publicly available datasets
- A list of figures that have associated raw data
- A description of any restrictions on data availability

The data that support the findings of this study are available from the corresponding author upon request (note that the data analyzed here is part of a longer undergoing study with a different aim and not yet published; as soon as the remaining parts of the data/study are completed, we will make our data available in a

public repository. In the meantime, we will make available the data included in this article upon request to the corresponding author). Source data are provided with this paper for Figures panels 2b and 4b.

Field-specific reporting

Please select the one below that is the best fit for your research. If you are not sure, read the appropriate sections before making your selection.

☐ Life sciences ☒ Behavioural & social sciences ☐ Ecological, evolutionary & environmental sciences

For a reference copy of the document with all sections, see nature.com/documents/nr-reporting-summary-flat.pdf

Behavioural & social sciences study design

All studies must disclose on these points even when the disclosure is negative.

Study description	Quantitative experimental study. This study aimed at developing a predictive machine learning model of stress responsiveness based on exploration behavior. More specifically, locomotion captured in ecologically valid virtual reality scenarios, inspired by the animal literature, developed in our lab. We further aimed at understanding wether these predictions generalize to different stressful scenarios experienced by a subset of participants: a 10 minute stress test in virtual reality based on a mental arithmetic task with social evaluation; a sustained anticipatory anxiety paradigm on a different day.
Research sample	Male students from the Swiss Federal Institute of Technology Lausanne (EPFL) and the University of Lausanne (UNIL). The mean age is 20.58 with a standard deviation of 2.05. The experiment was advertised at EPFL and UNIL and we asked for participants with ages between 18 and 38 years old and no history of psychiatric, cardiovascular health or motor related problems. The sample is then representative of healthy adult young males. We aimed at having a sample representative of young adults to reduce variability in the response to virtual reality. Note that the data analyzed here is part of a longer undergoing study with a different aim and not yet published.
Sampling strategy	Participants were sampled randomly from a pool of candidates that showed interest in participating in our experiment. No sample size calculation was performed since we wanted to to have enough samples for a learning and testing dataset. We split our sample of 135 participants into low, intermediate and moderately high trait-anxiety (STAI-T questionnaire) and used the low (STAI-T<35) and moderately high (STAI-T>45) for the training dataset to enhance the potential HRV variability in this set, and the intermediate group (35<=STAI-T<=45) for the test set. The number of independent samples in this study is in the same magnitude or larger than similar studies with dense data recording. Motion capture, physiology recording and questionnaire collection require long experiments which constrain the number of subjects that can be recruited. Furthermore, some heuristics can be used to calculate a sample size. For example, having n times more independent observations than model features. In this study, n is approximately 7.
Data collection	Questionnaire data was collected on computers using the Qualtrics platform privately and anonymously. Motion capture data was collected using the XSENS MVN Awinda system, position tracking data was collected using the VR headset HTC Vive coordinates and physiological data was collected with a Biopac system. All data was stored anonymously. The experimenter was blinded to the participants's information and details as well as the study's hypothesis, and was the only person running the experiment.
Timing	November 2016 to August 2018
Data exclusions	Due to missing EDA and pule data, 3 subjects were removed for the sustained anticipatory anxiety paradigm. Due to excessive movement artifact in the 2.5 minutes steps ECG, a total 8 subjects were removed for the statistics for physiology in Figure 4b.
Non-participation	No participant dropped or declined participating.
Randomization	The sample of 135 participants was split into low, intermediate and moderately high trait-anxiety (STAI-T questionnaire) and used the low (STAI-T<35) and moderately high (STAI-T>45) for the training dataset to enhance the potential HRV variability in this set, and the intermediate group (35<=STAI-T<=45) for the test set.

Reporting for specific materials, systems and methods

We require information from authors about some types of materials, experimental systems and methods used in many studies. Here, indicate whether each material, system or method listed is relevant to your study. If you are not sure if a list item applies to your research, read the appropriate section before selecting a response.

Materials & experimental systems

n/a	Involved in the study
☒	☐ Antibodies
☒	☐ Eukaryotic cell lines
☒	☐ Palaeontology and archaeology
☒	☐ Animals and other organisms
☐	☒ Human research participants
☒	☐ Clinical data
☒	☐ Dual use research of concern

Methods

n/a	Involved in the study
☒	☐ ChIP-seq
☒	☐ Flow cytometry
☒	☐ MRI-based neuroimaging

Human research participants

Policy information about studies involving human research participants

Population characteristics	Male participants with mean age of 20.58 with a standard deviation of 2.05. No history of psychiatric, cardiovascular health or motor related problems.
Recruitment	The experiment was advertised at the Swiss Federal Institute of Technology Lausanne (EPFL) and the University of Lausanne (UNIL) and we asked for participants with ages between 18 and 38 years old and no history of psychiatric, cardiovascular health or motor related problems. This recruitment might be affected by self-selection bias for participants who want to participate in virtual reality experiments, or that require monetary compensation. We aimed at having a sample representative of healthy adult young males and don't expect this self-selection bias to affect this aim.
Ethics oversight	Commission cantonale (VD) d'éthique de la recherche sur l'être humain (CER-VD)

Note that full information on the approval of the study protocol must also be provided in the manuscript.

This checklist template is licensed under a Creative Commons Attribution 4.0 International License, which permits use, sharing, adaptation, distribution and reproduction in any medium or format, as long as you give appropriate credit to the original author(s) and the source, provide a link to the Creative Commons license, and indicate if changes were made. The images or other third party material in this article are included in the article's Creative Commons license, unless indicated otherwise in a credit line to the material. If material is not included in the article's Creative Commons license and your intended use is not permitted by statutory regulation or exceeds the permitted use, you will need to obtain permission directly from the copyright holder. To view a copy of this license, visit http://creativecommons.org/licenses/by/4.0/
